# Serum Calcium and Magnesium Levels, Not 25-Hydroxyvitamin D, Are Associated With Sleep Quality in Airline Pilots

**DOI:** 10.7759/cureus.50940

**Published:** 2023-12-22

**Authors:** Piercarlo Minoretti, Andrés Santiago Sáez, Ángel García Martín, Miryam Liaño Riera, Manuel Gómez Serrano, Enzo Emanuele

**Affiliations:** 1 Occupational Health, Studio Minoretti, Oggiono, ITA; 2 Legal Medicine, Hospital Clinico San Carlos, Madrid, ESP; 3 Legal Medicine, Psychiatry, and Pathology, Complutense University of Madrid, Madrid, ESP; 4 Occupational Health, 2E Science, Robbio, ITA

**Keywords:** magnesium, calcium, vitamin d, pittsburgh sleep quality index, sleep quality, airline pilots

## Abstract

Introduction: Despite the vulnerability of airline pilots (APs) to sleep disturbances, the biological underpinnings responsible for this phenomenon are still not entirely elucidated. However, there is an increasing amount of evidence indicating an association between 25-hydroxyvitamin D, Ca^2+^, and Mg^2+ ^levels and sleep health. In this cross-sectional study, we sought to examine the potential associations between serum levels of these biomarkers and the occurrence of poor sleep among APs.

Methods: We examined a convenience sample of 100 male APs who underwent the Pittsburgh Sleep Quality Index (PSQI) to assess their sleep quality. Those who scored 5 or higher on the PSQI were labeled as poor sleepers. Serum levels of 25-hydroxyvitamin D, Ca^2+^, and Mg^2+^ were quantified in all participants.

Results: Out of the 100 APs, 58 (58%) and 42 (42%) were classified as good and poor sleepers, respectively, based on the PSQI scores. We defined vitamin D deficiency as serum 25-hydroxyvitamin D levels below 10 ng/mL and insufficiency as levels ranging from 10 to 30 ng/mL. The results revealed no significant differences in serum levels of 25-hydroxyvitamin D between the two groups, and there was no evidence of vitamin D deficiency or insufficiency. However, poor sleepers exhibited significantly lower levels of both Mg^2+ ^(1.8 ± 0.1 mg/dL versus2.0 ± 0.1 mg/dL, respectively) and Ca^2+^ (8.5 ± 0.4 mg/dL versus9.1 ± 0.5 mg/dL, respectively) compared to good sleepers (*P* < 0.001 for both). Logistic regression analysis identified both Mg^2+^ and Ca^2+^ as independent biomarkers associated with poor sleep quality in APs (*P* < 0.001 for both).

Conclusion: Lower serum concentrations of Mg^2+^ and Ca^2+^, not 25-hydroxyvitamin D, may be associated with poor sleep in APs.

## Introduction

In 1973, Preston was the first to document a high prevalence of sleep disturbances among airline pilots (APs) [1], a professional group particularly vulnerable to circadian desynchronization [2] and fatigue [3]. These initial findings paved the way for subsequent investigations in the field, which have continually substantiated this correlation [4-6]. Among a large sample of 435 APs, Reis et al. [5] reported prevalence rates of sleep complaints and daytime sleepiness at 34.9% and 59.3%, respectively. In a study of 332 long-haul APs, Wu et al. [7] found that pilots experienced sleep restriction and circadian disruption during their flights. Moreover, when compared to age-matched individuals from the general population, their baseline sleep duration, as determined by actigraphy, was notably longer [7]. Marqueze et al. [8] investigated 1,235 Brazilian APs operating national or international flights and found a prevalence of 57.8% for unintentional sleep while flying. Similarly, in another study of 1,234 APs, Pellegrino et al. [9] reported a prevalence of 48.2% for poor sleep quality. In a recent study conducted in 344 pilots, half the sample was found to be at risk for insomnia and fatigue [2]. Furthermore, 59 (17.2%) pilots were identified as being at high risk for sleep apnea [2]. A separate study by Han et al. [10] involved 103 pilots who underwent daytime polysomnography following a long-haul night-time flight. Results showed that 73 participants (70.9%) exhibited moderate-to-severe obstructive sleep apnea (OSA) [10]. Despite the published wealth of research, the biological mechanisms underlying disrupted sleep in APs remain poorly understood. However, from a biological standpoint, it has been observed that shorter sleep duration in pilots employed on early shifts is associated with a higher cortisol increase in response to awakening, a greater total cortisol output throughout the day, and a slower rate of decline over the day [11].

There is a growing body of evidence suggesting a significant correlation between circulating vitamin D and sleep quality [12]. The importance of sleep biomarkers, such as vitamin D, lies in their potential to serve as indicators for the identification and management of sleep disorders. Vitamin D deficiency may increase the likelihood of developing sleep disorders, lower the quality, shorten the duration, and generally disrupt sleep patterns [13]. In the field of occupational medicine, a recent study conducted on 1,423 male shift workers found a higher occurrence of OSA in individuals with vitamin D deficiency [14]. Interestingly, magnesium (Mg^2+^), a vital component in various biochemical processes, plays a crucial role in the synthesis and metabolism of vitamin D [15]. The conversion of inactive 25-hydroxyvitamin D to its active form, 1,25-dihydroxyvitamin D, is dependent on magnesium [15]. Importantly, Mg^2+^ directly influences sleep mechanisms by regulating the glutamatergic and gamma-aminobutyric acid (GABA) ergic system, ultimately reducing nervous system excitability [16]. Furthermore, Mg^2+^ inhibits intracellular calcium (Ca^2+^) concentration within the muscle, thus promoting muscle relaxation by suppressing the N-methyl-D-aspartate receptor [16]. Research on rats has also demonstrated a significant association between magnesium deficiency and a decrease in plasma melatonin, a well-known sleep-promoting hormone [17]. Both vitamin D and Mg^2+^ are closely intertwined with Ca^2+^ homeostasis, which is responsible for regulating various physiological functions, including sleep [18]. Calcium fluctuations and intracellular potentials have been found to govern cortical slow-wave oscillations, which are the hallmark of slow-wave sleep [18]. Furthermore, a correlation between Ca^2+^ levels and sleep latency, as well as non-restorative sleep, has been recently reported [19].

Starting from these premises, we designed the current cross-sectional study to investigate the relationship between serum levels of 25-hydroxyvitamin D, Ca^2+^, Mg^2+^, and the presence of poor sleep in APs. The hypothesis was that individuals with varying sleep qualities would demonstrate significantly different levels of specific biomarkers. Considering that pilots are responsible for the safety of hundreds of passengers and crew members, and their vigilance is crucial for ensuring flight safety and efficiency, our study could provide valuable insights into the biological foundations of sleep for this professional group. In addition, our findings may lead to the development of strategies to manage sleep more effectively.

## Materials and methods

Participants

A total of 100 male APs of Caucasian descent volunteered to participate in the study. They were recruited during routine occupational health visits at outpatient clinics (Studio Minoretti S.r.l., Oggiono, Italy), with an occupational health physician extending the invitation [20]. Due to the limited number of female pilots, women were excluded. Eligibility criteria included the absence of endocrine, psychiatric, neurological, autoimmune, or infectious diseases. Participants with a known history of OSA, cancer, or recent medication use within the past 90 days were also ineligible. None of the participants reported taking dietary supplements, including vitamin D and mineral supplements, and all were observed to be in good physical health. The study adhered to the tenets of the Declaration of Helsinki, and the local ethics committee approved the study protocol (Studio Minoretti, reference number: 2021/04E). Written informed consent was obtained from all participants.

Sleep quality

The sleep quality of APs was evaluated using the Pittsburgh Sleep Quality Index (PSQI), a validated 19-item self-reported questionnaire [21]. The PSQI has been extensively described in the literature and has demonstrated strong clinical and psychometric properties. This questionnaire comprises 19 items and produces a global score that ranges from 0 to 21, with higher scores indicating worse sleep quality. A global score exceeding 5 suggests that the person is a poor sleeper [22]. The PSQI has a sensitivity of 89.6% and specificity of 86.5% in identifying individuals with sleep disorders, using the standard cutoff score of 5 [22].

Laboratory methods

Venous blood samples were collected through venipuncture after an overnight fast using exclusively polypropylene tubes to prevent metal contamination. The blood samples were then centrifuged at 3,000 rpm for 15 minutes in a dust-free room to obtain the sera, which was subsequently stored at -20°C until further analysis. Circulating levels of 25-hydroxyvitamin D were determined using an Accubind commercial ELISA kit (Monobind Inc., Lake Forest, CA, USA) according to the manufacturer’s instructions. The minimum detection limit was 1.14 ng/mL, and the inter- and intra-assay coefficients of variation were less than 9% and 7%, respectively. The samples were randomized within different batches, and the laboratory staff was uninformed of the participants’ PSQI scores. Each subject's sample was double-analyzed in the same assay, with results subsequently averaged. The assay guidelines classified vitamin D deficiency as serum 25-hydroxyvitamin D levels below 10 ng/mL, insufficiency as levels ranging from 10 to 30 ng/mL, sufficiency as levels within 31 and 100 ng/mL, and toxicity as levels exceeding 100 ng/mL. The total concentrations of Mg^2+^ and Ca^2+^ were determined using a Hitachi 7700 automatic analyzer (Hitachi, Tokyo, Japan). The normal reference ranges for Mg^2+^ and Ca^2+^ in our laboratory are 1.7-2.2 mg/dL and 8-10 mg/dL, respectively.

Data analysis

We divided the sleep quality of our sample into two groups using the standard cut-off score of 5 for the PSQI [21]. Pilots who scored between 0 and 4 were classified as “good sleepers,” whereas those with a score of 5 or higher were labeled as “poor sleepers.” To evaluate the normality of continuous data, we utilized the Kolmogorov-Smirnov test. The results confirmed that all variables followed a normal distribution, which justified the sole use of parametric statistical methods. Continuous data are presented as the mean ± standard deviation, while categorical data are given as counts and percentages. To compare variables between good and poor sleepers, the Student’s t-test was used for continuous data, whereas the chi-squared test was employed for categorical variables. The Pearson’s correlation coefficient was utilized to assess the correlations between continuous variables. We employed logistic regression analysis, utilizing the forward conditional selection method, to identify a combination of biomarkers that could potentially correlate with poor sleep in APs. The predicted probability value was derived from each logistic regression model. In addition, we conducted a leave-one-out cross-validation to evaluate the generalizability of our equation. Data analyses were conducted using the computer software package IBM SPSS Statistics for Windows, version 20 (released 2011; IBM Corp., Armonk, New York, United States). To mitigate the likelihood of type I statistical errors, the Bonferroni correction was applied. This strategy adjusts the threshold P value by dividing the standard 0.05 significance level by the total number of comparisons. Given that our study focused on three distinct biomarkers, the number of comparisons was three, resulting in a threshold P value of 0.05/3 = 0.017.

## Results

In our study sample consisting of 100 APs, we found that 58% (n = 58) fell into the category of good sleepers, exhibiting a PSQI score between 0 and 4. By contrast, the remaining 42% (n = 42) were identified as poor sleepers, as their scores surpassed the cutoff of 5. Table 1 summarizes the general characteristics, including serum levels of 25-hydroxyvitamin D, Mg^2+^, and Ca^2+^, of the two groups.

**Table 1:** General characteristics of airline pilots categorized according to the quality of sleep Abbreviations: BMI, body mass index; ns, not significant

	Good sleepers (n = 58)	Poor sleepers (n = 42)	P
Age, years	41 ± 4	40 ± 5	ns
Male sex, n (%)	58 (100)	42 (100)	ns
BMI, kg/m^2^	24 ± 3	23 ± 4	ns
Education, years	17 ± 3	17 ± 4	ns
25 hydroxyvitamin D, ng/mL	38.9 ± 4.2	39.2 ± 4.9	ns
Mg^2+^, mg/dL	2.0 ± 0.1	1.8 ± 0.1	<0.001
Ca^2+^, mg/dL	9.1 ± 0.5	8.5 ± 0.4	<0.001

Age, body mass index, education, and 25-hydroxyvitamin D levels exhibited no significant intergroup differences. Importantly, none of the APs in the study showed either a deficiency or insufficiency of vitamin D, defined as 25-hydroxyvitamin D levels below 10 ng/mL or between 10 and 30 ng/mL, respectively.

Despite the Mg^2+^ and Ca^2+^ levels in all APs falling within the reference ranges, those classified as poor sleepers exhibited significantly lower levels of both Mg^2+^ (1.8 ± 0.1 mg/dL compared to 2.0 ± 0.1 mg/dL) and Ca^2+^ (8.5 ± 0.4 mg/dL compared to 9.1 ± 0.5 mg/dL) when compared with good sleepers (P < 0.001 for both; Table 1). Moreover, significant negative correlations were observed between Mg^2+^ (r = −0.37, P = 0.002) and Ca^2+^ (r = −0.30, P = 0.009) and PSQI scores across the entire study sample. Logistic regression analysis (Table 2) identified both Mg^2+^ and Ca^2+^ as independent markers associated with poor sleep quality in APs (P < 0.001 for both). The equation formulated by the analysis for predicting poor sleep is as follows: predicted probability = 1/[1 + 1/exp (1.475 − 0.005 × (Mg^2+^ ) − 0.031 × (Ca^2+^)].

**Table 2:** Logistic regression analysis indicating independent associations between serum Mg2+ and Ca2+ levels and poor sleeping in airline pilots

	Coefficient (beta)	Odds ratio	95% confidence interval	P
Mg^2+^, mg/dL	-0.005	0.992	0.899−0.996	<0.001
Ca^2+^, mg/dL	-0.031	0.995	0.992−0.997	<0.001
Constant	1.475	-	-	0.22

## Discussion

Our findings indicate that 42% of our APs experienced poor sleep, a percentage that is consistent with the previously reported rates of 34.9% [5] to 48.2% [9] found in larger studies. However, this cross-sectional investigation represents a pioneering effort in uncovering the variations in serum biomarkers between APs who suffer from inadequate sleep compared to those with optimal sleep routines. Specifically, the study reveals three key findings. First, APs, in general, exhibited sufficient levels of 25-hydroxyvitamin D without any signs of deficiency or insufficiency. Second, we did not find any significant correlations between serum 25-hydroxyvitamin D levels and poor sleep. Lastly, we observed lower concentrations of Mg^2+^ and Ca^2+^ in APs classified as poor sleepers, suggesting a potential link between these elements and disrupted sleep. These findings were further supported by logistic regression analysis, which indicated an inverse independent association between Mg^2+^ and Ca^2+^ levels and sleep quality among APs.

The absence of significant differences between poor and good sleepers with regard to serum 25-hydroxyvitamin D concentrations seems to suggest that they are unlikely to play a major role in influencing sleep in healthy APs. These results differ from previously reported findings in the general population [12,13] and shift workers [14], wherein vitamin D emerged an independent predictor of sleep efficiency. However, one notable difference between these studies and our current research is that we did not observe any evidence of vitamin D deficiency or insufficiency in our sample of APs. Our findings support the conclusions of Lee et al. [23], who observed no significant correlation between serum vitamin D levels and sleep parameters in night-shift workers. However, their study did reveal a strong association between vitamin D deficiency and a reduced duration of total sleep time in daytime workers [23]. This implies that the negative impact on sleep quality may be linked to vitamin D deficiency or insufficiency, rather than merely lower values within the normal reference range. In a recent study conducted by Morais-Moreno et al. [24], it was found that the mean 25-hydroxyvitamin D levels in 235 Spanish APs aged between 22 and 65 years were below the lower limit of normal (30 ng/mL), with a mean concentration of 28.9 ± 8.2 ng/mL. While the exact reasons for the disparities between this study and the current research remain unclear, they could potentially be attributed to variations in dietary habits, work schedules, and sun exposure during leisure activities. Although we did not observe any significant differences in serum concentrations of 25-hydroxyvitamin D between poor and good sleepers in our APs, it is important to interpret these findings cautiously. To reinforce our results, it will be essential to incorporate objective measures of sleep quality, such as total sleep duration, sleep architecture, wakefulness during sleep episodes, and the frequency and duration of awakenings.

Contrary to 25-hydroxyvitamin D, we observed that APs with poor sleep quality exhibited lower serum levels of Mg^2+^ and Ca^2+^ compared to good sleepers, despite both elements being within normal reference ranges. The correlation between Mg^2+^ status and sleep quality within our research sample of APs aligns with previous observational studies conducted on the general population, as reviewed recently by Arab and coworkers [25]. Notably, sleep deprivation appears to decrease Mg^2+^ levels in patients suffering from OSA [26], a condition commonly observed among APs [10]. In addition, Mg^2+^ levels tend to improve after treatment, mirroring the reduction in OSA severity [26]. A randomized controlled trial involving 46 elderly individuals with insomnia found that magnesium supplementation improved sleep quality [27]. The study divided participants into two groups, both with similar initial serum magnesium levels. The group that received magnesium supplementation showed enhanced sleep parameters, which were associated with increased serum magnesium levels, in contrast to the placebo group [27]. Nevertheless, our findings should not be interpreted as an endorsement for Mg^2+^ supplementation to improve sleep in APs, particularly in persons who initially have a normomagnesemic status.

In a recent study conducted in shift workers, Jeon et al. [19] reported a negative correlation between Ca^2+^ levels and various sleep parameters, including sleep latency, total sleep time, reliance on sleep medication, and daytime dysfunction. Our research echoes these findings, as it highlights an inverse, independent link between Ca^2+^ concentrations and the PSQI score among APs. In a comprehensive study involving 1422 young adults, Alkhatatbeh et al. [28] identified an association between low calcium intake and the onset of sleep disorders, as well as anxiety, depression, and musculoskeletal pain. However, given the tightly controlled regulation of Ca^2+^ levels and its pervasive involvement in nearly all neural activities, it is crucial to conduct more extensive research on whether calcium supplementation can ultimately enhance sleep quality in APs. In this context, it may also be beneficial to focus on sleep-regulating kinases, such as the Ca^2+^/calmodulin-dependent protein kinase II, as a potential strategy to manage sleep-related functions [18].

While our research provides intriguing preliminary findings, it is essential to acknowledge several limitations. First, this study did not obtain objective sleep quality metrics as this fell beyond the scope of standard occupational medicine consultations. Likewise, the study did not meticulously probe the occurrence of OSA. Despite the sex distribution within our sample accurately reflecting the broader demographic of APs, the lack of female participants do actually restrict the generalizability of our results to women. Therefore, there is a critical need for more comprehensive research involving a larger, more diverse sample size to substantiate and expand our conclusions. Finally, our research was cross-sectional in design. As a result, while we could identify relationships, we were unable to definitively confirm prediction or causation.

## Conclusions

Despite these limitations, we found an independent association between poor sleep among APs and lower serum concentrations of Mg^2+^ and Ca^2+^. Intriguingly, this correlation was not detected in relation to 25-hydroxyvitamin D. Although these observations imply that serum levels of Mg^2+^ and Ca^2+ ^may potentially influence sleep quality and duration among these professionals, the precise nature and magnitude of this impact require further exploration and understanding. It remains to be determined if supplementing Mg^2+^ and Ca^2+^ could be a feasible approach to enhance sleep among APs whose serum levels of these elements fall within the reference range.
